# FAM209 associates with DPY19L2, and is required for sperm acrosome biogenesis and fertility in mice

**DOI:** 10.1242/jcs.259206

**Published:** 2021-11-01

**Authors:** Julio M. Castaneda, Keisuke Shimada, Yuhkoh Satouh, Zhifeng Yu, Darius J. Devlin, Masahito Ikawa, Martin M. Matzuk

**Affiliations:** 1 Research Institute for Microbial Diseases, Department of Experimental Genome Research, Osaka University, Osaka 5620031, Japan; 2 Institute for Molecular and Cellular Regulation, Department of Molecular and Cellular Biology, Gunma University, Gunma 3718512, Japan; 3 Department of Pathology & Immunology and Center for Drug Discovery, Baylor College of Medicine, Houston, TX 77030, USA

**Keywords:** Dpy19l2, Fam209, Acrosome, Spermiogenesis

## Abstract

Infertility afflicts up to 15% of couples globally each year with men a contributing factor in 50% of these cases. Globozoospermia is a rare condition found in infertile men, which is characterized by defective acrosome biogenesis leading to the production of round-headed sperm. Here, we report that family with sequence similarity 209 (*Fam209*) is required for acrosome biogenesis in mouse sperm. FAM209 is a small transmembrane protein conserved among mammals. Loss of *Fam209* results in fertility defects that are secondary to abnormalities in acrosome biogenesis during spermiogenesis, reminiscent of globozoospermia. Analysis of the FAM209 proteome identified DPY19L2, whose human orthologue is involved in the majority of globozoospermia cases. Although mutations in human and mouse *Dpy19l2* have been shown to cause globozoospermia, no *in vivo* interacting partners of DPY19L2 have been identified until now. FAM209 colocalizes with DPY19L2 at the inner nuclear membrane to maintain the developing acrosome. Here, we identified FAM209 as the first interacting partner of DPY19L2, and the second protein that is essential for acrosome biogenesis that localizes to the inner nuclear membrane.

## Abstract (precis)

**Summary:** We report that a conserved novel gene, *Fam209*, is essential for sperm development in the mouse. The phenotype seen in the absence of *Fam209* mimics that of defective sperm in some infertile men.

## INTRODUCTION

Infertility is the inability to conceive after one year of unprotected sex and afflicts up to 15% of couples worldwide (Thonneau et al., 1991; World Health Organization, 2018; CDC National Survey of Family Growth, https://www.cdc.gov/nchs/nsfg/key_statistics/i_2015-2017.htm). Fertility problems on the male side contribute to 50% of infertility cases (Agarwal et al., 2015). Among infertile men, globozoospermia, i.e. round-headed sperm, is a rare condition characterized by defects in acrosome biogenesis during spermiogenesis (Kullander and Rausing, 1975; Schirren Sen et al., 1971). The acrosome is a large, sperm-specific organelle filled with degradative enzymes and receptors that facilitate sperm–egg interactions (Fléchon, 2016; Okabe, 2016). Although acrosome formation has been described at light- and electron-microscope levels for decades, the molecular mechanisms of acrosome formation are still being elucidated (Khawar et al., 2019; Leblond and Clermont, 1952). In mammals, the acrosome begins to form after meiosis when germ cells enter the haploid round spermatid stage (Russell et al., 1990). Acrosome vesicles derived from the trans-Golgi network accumulate and fuse adjacent to the nucleus to form a cap-like structure (Ho et al., 1999). During spermiogenesis, the acrosome cap then matures in coordination with other cellular processes to acquire its final shape in the mature sperm depending on the species, i.e. pear-shaped in humans and hook-shaped in many rodents. Golgi-associated and PDZ coiled-coil domain containing protein (GOPC) (Ito et al., 2004; Neudauer et al., 2001; Yao et al., 2002), protein interacting with C Kinase 1 (PICK1) (Staudinger et al., 1995; Xiao et al., 2009), and serine-rich single-pass transmembrane protein 1 (SSMEM1) (Nozawa et al., 2020) have been shown to be essential for trafficking vesicles from the Golgi complex to the developing acrosome. Zona pellucida-binding protein 1 (ZPBP1) (Lin et al., 2007) and sperm acrosome membrane-associated 1 (SPACA1) (Fujihara et al., 2012; Mori et al., 1993) localize to the acrosome membrane and are required for acrosome maturation. The cytoskeletal proteins F-actin and keratin 5 contribute to formation of a complex called the acroplaxome, an electron-dense structure that is sandwiched between the inner acrosome membrane and the outer nuclear envelope, which anchors the acrosome near the nucleus (Kierszenbaum et al., 2003). DPY19L2 was the first inner nuclear envelope protein shown to be essential for anchoring the acrosome to the nucleus and has been implicated in globozoospermia in the majority of patients analyzed (Koscinski et al., 2011; Pierre et al., 2012). Here, we report the discovery of FAM209 as the second inner nuclear membrane protein required for acrosome biogenesis. We show that mouse FAM209 is a conserved, transmembrane, testis-specific protein expressed in the haploid phase of spermatogenesis when the acrosome is developing. FAM209 localized to the inner nuclear envelope and interacted with DPY19L2. In this report, we characterized two deleterious mutations in mouse *Fam209* generated by CRISPR/Cas9 and propose that *Fam209* is essential for acrosome biogenesis.

## RESULTS

### Murine *Fam209* is a conserved and testis-enriched gene

**Fig. 1.**
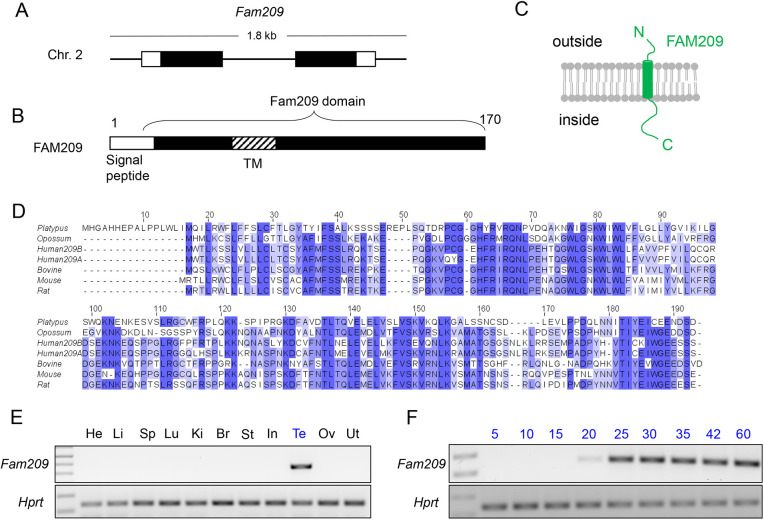
**FAM209 is conserved and predominately expressed in testis.** (A) Schematic of the mouse *Fam209* locus. (B) Schematic of FAM209 protein with the signal peptide, transmembrane domain, and FAM209 domain indicated. (C) Predicted topology of mouse FAM209. (D) Conservation of the amino acid sequence of FAM209 proteins from several mammalian species. Human FAM209A and B are included. (E) RT-PCR analysis of *Fam209* expression from various mouse tissues; expression of *Hprt* was used as control. He, heart; Li, liver; Sp, spleen; Lu, ling; Ki, kidney; Br, breast; St, stomach; In, intestines; Te, testis; Ov, ovaries; Ut, uterus. (F) RT-PCR analysis of *Fam209* expression from postnatal testis obtained at minutes postnatal as indicated; expression of *Hprt* was used as control.

*Fam209* in mouse is a testis-expressed gene located on chromosome 2 that encodes a 170 amino acid (aa) transmembrane precursor protein (Fig. 1A,B). Phobius software analysis identifies a cleaved signal peptide from aa 1–20 and a transmembrane domain from aa 40–60 (Fig. S1A) (Käll et al., 2004, 2007). The mature protein has been predicted to be 150 aa long, with the N-terminal part of FAM209 outside and the C-terminal part inside the cell (Fig. 1C). FAM209 orthologues are only present in all three branches in mammals (monotremes, marsupials, and eutherians) and absent in other taxa (Fig. S1B). Sequence alignment of FAM209 orthologues showed high conservation along most of the protein; this region of high conservation among FAM209 orthologues was designated as the FAM209 domain (Fig. 1D). The function of this domain is unknown. The gene underwent a duplication event in the human lineage that contains two paralogs, *FAM209A* and *FAM209B*. Human FAM209A and FAM209B are 92% similar between themselves, but both show 74% similarity to mouse FAM209. When using RT-PCR analysis of mouse tissues, we detected expression of *Fam209* only in testis, with expression detectable at postnatal day 20 when round spermatids begin to appear (Fig. 1E and F). Expression databases indicate that human *FAM209A/B* expression is predominately in testis (Fagerberg et al., 2014).

### *Fam209* is required for male fertility

**Fig. 2.**
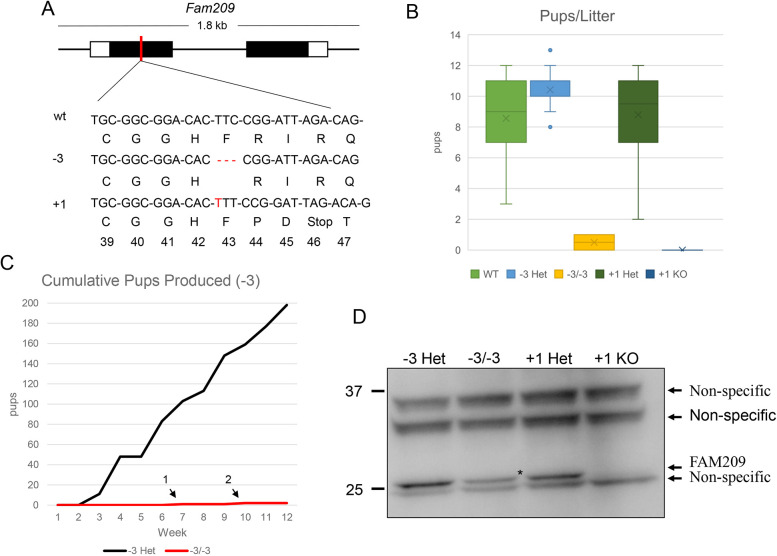
***Fam209* is important for male fertility.** (A) Mutation of *Fam209* using CRISPR/Cas9. Shown are the deletion of three thymine residues (−3) yielding a FAM209 that lacks the phenylalanine at amino acid position 43, and the insertion of one thymine nucleotide (+1) yielding the +1 frameshift mutant that comprises a STOP codon. (B) Results from breeding tests using males of the indicated genotype after pairing with wild-type (wt) females for at least 8 weeks. Data are the average number of pups per litter; *n*=3 for heterozygous mice (–3 Het, +1 Het and +1 KO), *n*=5 for homozygous (−3/−3 mice). Boxes show the interquartile range (IQR) and whiskers mark 1.5×IQR. Median values are marked by a line and mean values are indicated by crosses. (C) Cumulative number of pups produced from −3 allele heterozygous (*n*=3) and homozygous (*n*=5) males. The two numbered arrows indicate the pups sired by −3/−3 mice (at weeks 7 and 10, respectively). (D) Western blotting for FAM209 of testis lysates from −3 Het, −3/−3, +1 Het and +1 KO mice. Lysate from −3/−3 mice shows a 25-kDa FAM209 band (asterisk). Testis lysate from +1 KO mice do not show the 25 kDa band. −3 Het, *Fam209*^*+/−3*^mice; −3/−3, *Fam209^−3/−3^* mice; +1 Het, *Fam209*^*+/*+*1*^ mice; +1 KO, *Fam209^+1/+1^* mice.

To understand the roles of *Fam209*, we used CRISPR/Cas9 to generate random indel mutations in mouse *Fam209*. With this method, two *Fam209* alleles were obtained. The first comprises an in-frame deletion of three thymine nucleotides leading to deletion of phenylalanine at position 43 (–3 or *Fam209^−3^*). The second comprises the insertion of an additional thymine residue at position 129 (+1 or *Fam209^+1^*) within the coding sequence, causing a frameshift mutation (Fig. 2A and Fig. S1C). Homozygous mice for either allele did not display any overt abnormalities. To test fertility, homozygous males were co-housed with wild-type females for 8 weeks, which were monitored for plug formation and birth of pups. Wild-type females and −3 and +1 heterozygous males produced pups, but +1 knockout (KO) males did not sire any pups despite plug formation (*n*=3 for each genotype) (Fig. 2B). However, one out of five −3 homozygous mutant males sired only two pups (Fig. 2C). Females homozygous for either the −3 or +1 allele were fertile. Using western blot analysis, we detected FAM209 in lysates of testes from the heterozygous controls and that it is completely absent in +1 KO mice; however, we still observed FAM209 in the −3 homozygous mutant males (Fig. 2D). These data implicate mouse *Fam209* being essential for male fertility and suggest that the −3 allele is a hypomorphic allele compared with the +1 allele.

To understand why *Fam209* mutant males are less fertile, we first examined sperm quality by using computer-assisted sperm analysis (CASA). Sperm from control and homozygous mutant adults was collected from the cauda epididymis and incubated in capacitation medium for 10 min. After incubation, sperm were analyzed with CASA, showing that, on average, <10% of sperm derived from −3 or +1 homozygous mice were motile (Fig. S2A). Analysis of the few motile sperm showed that the average path, straight-line and curvilinear velocities were decreased, which is indicative of decreased sperm motility (Fig. S2B). We also checked the morphology of isolated sperm and saw abnormally shaped sperm heads in –3 and +1 mutant mice, reminiscent of globozoospermia phenotypes observed in other mouse KOs such as *Dpy19l2* and *Gopc* (Fig. S2C). These data indicate that *Fam209* is required for proper sperm formation.

### FAM209 is required for acrosome biogenesis

**Fig. 3.**
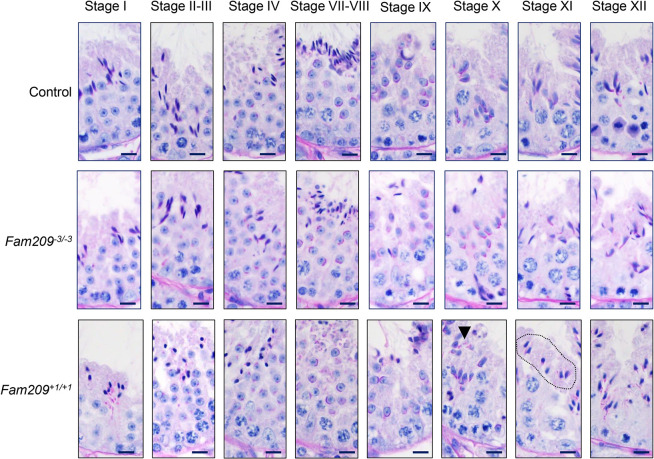
**Expression of the +1 *Fam209* allele yields a more-severe phenotype than expression of the **−3** ***Fam209*** allele.** Representative of PAS-stained microscopy images of testis cross sections from control, *Fam209^−3/−3^*, *Fam209^+1/+1^* mice at several stages of spermatogenesis. *Fam209^−3/−3^* spermatid progression is grossly comparable to that of control mice. PAS staining of *Fam209^+1/+1^* testis first detected abnormal localization of acrosome contents (dark pink staining, arrowheads) at stage X. Abnormally shaped elongating spermatids are encircled in *Fam209^+1/+1^* stage XI images (dashed line). Scale bars: 10 µm.

To explore at which point mutant males begin to show abnormalities regarding the production of sperm, we performed histology and immunofluorescence analysis on testis sections. Periodic acid–Schiff (PAS) staining on testis cross sections obtained from control mice revealed various stages of spermatogenesis (Russell et al., 1990) in seminiferous tubules (Fig. S2D). Similarly, the −3 mutant testes displayed apparently normal spermatogenesis by using PAS staining. In the +1 KO mouse, histology identified defects in elongating spermatids, in the form of highly condensed nuclei. Upon closer examination, analysis of +1 KO identified the transition from step 9 to step 10 of the development of spermatids (Russell et al., 1990) when a defect is readily observable in PAS stains. Defects in the +1 KO also include aberrant staining of the acrosome (dark pink signal) in addition to the compacted nuclei (Fig. 3). To further examine the acrosome, immunofluorescence was performed against the two acrosomal membrane proteins IZUMO1 and SPACA1 (Fig. S3). IZUMO1, a protein required for sperm-egg fusion, is located throughout the acrosome membrane in round spermatids and gets restricted to the acrosome cap in elongated spermatids (Inoue et al., 2005; Satouh et al., 2012). SPACA1, a protein required for acrosome formation, is located in the inner acrosomal membrane within round spermatids and in the equatorial segment of the acrosome in elongated spermatids (Fig. S3A and B) (Fujihara et al., 2012; Hao et al., 2002). Elongating spermatids of the −3 mutant show very subtle differences in SPACA1 localization compared to that of control mice (Fig. S3C and D). Severe defects in IZUMO1 and SPACA1 localization are seen in the +1 KO spermatids, where both components appear to be not adjacent to the nucleus (Fig. S3E and F). In addition, compacted nuclei were also seen by staining with Hoechst 33342. Transmission electron microscopy (TEM) revealed subtle defects in the −3 allele compared to control, with the acrosome cap showing slight morphology changes during the cap phase of acrosome development, including protrusions near the acrosome granule (Fig. S3G and J). In elongated spermatids from −3 mutants, there appeared to be a large gap between the electron-dense components of the acrosome and the nucleus when compared to spermatids from control mice (Fig. S3I and K). TEM analysis of the +1 KO mice showed aberrant localization of acrosome dense material in step 9 spermatids and acrosome loss in some late-stage spermatids (Fig. S3L and M). These data further demonstrated that the −3 allele is hypomorphic compared to the +1-frameshift mutation and that full-length FAM209 is required for acrosome biogenesis.

### FAM209 localizes to the inner nuclear envelope

**Fig. 4.**
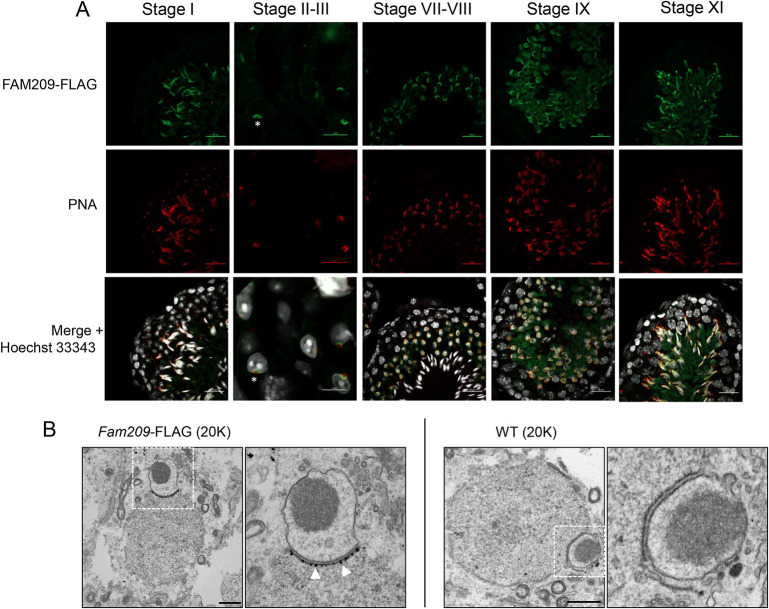
**FAM209 localizes to the inner nuclear envelope beneath the acrosome.** (A) FLAG-immunostaining of testis from *Fam209^Flag/Flag^* mice, showing FAM209-FLAG located near the acrosome (green). Peanut agglutinin antibody (PNA) staining (red) indicates the developing acrosome. FAM209-FLAG starts to be expressed at stages II–III (asterisk). Scale bars: 20 μm for stage I, VII-VIII, IX and XI; 5 μm for stage II-III. (B) Immunogold labeling of FLAG in ultra-thin testis sections obtained from *Fam209^Flag/Flag^* and control mice. Dashed boxes indicate regions shown in enlarged images on the right. Arrowheads indicate gold particles in the inner nuclear membrane of spermatids. Scale bars: 1 µm.

To begin to understand the role of FAM209 in biogenesis of the acrosome, we examined its localization during spermatogenesis. Unfortunately, the rabbit polyclonal antibody developed for FAM209 western blots was unsuitable for immunofluorescence. To circumvent this problem, we used FLAG-tagged *FAM209* to generate a FAM209-FLAG knock-in mouse (*Fam209^Flag/Flag^*) (Fig. S4A) by adding the FLAG epitope sequence to the C-terminus of the endogenous *Fam209* locus (Fig. S4B). *Fam209^Flag/Flag^* mice were fertile, suggesting that the FLAG epitope does not interfere with FAM209 function. Using anti-FLAG antibodies to detect the FAM209 fusion protein, we detected expression in stage II–III seminiferous tubules as determined by PNA (peanut-agglutinin antibody) staining (Fig. 4A). In stages II–III, the acrosome granule, labeled with PNA, contacted the nucleus, and FAM209-FLAG staining appeared underneath the PNA signal. FAM209-FLAG signal was present in the region of the developing acrosome through most of spermiogenesis until step 16 when spermiation begins. The initial FAM209-FLAG staining pattern underneath the PNA signal in step 2–3 spermatids suggests that FAM209 localized to beneath the acrosome; however, PNA preferentially binds the outer acrosomal membrane (Mortimer et al., 1987), which left the possibility that FAM209 can localize to the inner acrosomal membrane or to the nuclear membrane. We used additional acrosomal membrane markers IZUMO1 and SPACA1 to determine whether FAM209 is, indeed, located beneath the acrosome. IZUMO1 has a uniform distribution around the acrosome, whereas SPACA1 localizes to the inner acrosomal membrane (Hao et al., 2002; Inoue et al., 2005). In both double stains, the FAM209-FLAG signal was located underneath IZUMO1 and SPACA1 (Fig. S4C and D, Movies 1 and 2). To further confirm our findings, we performed immuno-electron microscopy (EM) using gold-labeled antibodies. Ultra-thin testis sections obtained from *Fam209^Flag/Flag^* mice and stained with a rabbit polyclonal antibody against FLAG showed gold particles inside the nucleus within the region of the acrosome, whereas these particles were absent in the wild-type control (Fig. 4B and Fig. S4E). The observed non-specific localization of gold particles in control and *Fam209^Flag/Flag^* mouse samples might be due to the polyclonal nature of the FLAG antibody (Fig. S4F). These staining patterns further indicated that FAM209 is located underneath the developing acrosome and, specifically, at the inner nuclear membrane.

### FAM209 associates with globozoospermia-associated protein DPY19L2

**Fig. 5.**
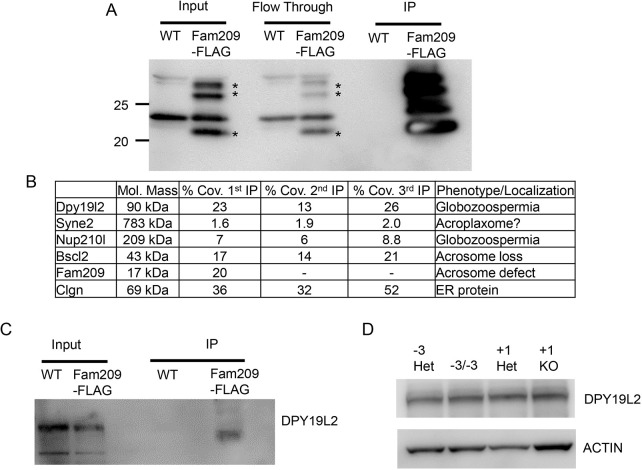
**FAM209 immunoprecipitation identifies DPY19L2.** (A) Western blot analysis of immunoprecipitated testis lysate samples from mice carrying a FLAG-tagged allele of FAM209. Asterisks highlight bands specific to the *Fam209^Flag/Flag^* knock-in mice (Fam209-FLAG) that are absent in wild-type (WT) control. (B) Mass spectrometry results from three independent immunoprecipitations of FAM209-FLAG from testis lysates. The coverage (expressed as a percentage; % Cov.), expression in testis and phenotype/localization are provided. (C) Western blot analysis of FAM209-FLAG immunoprecipitation. The inner nuclear membrane protein DPY19L2 co-immunoprecipitates with FAM209-FLAG. (D) Western blot analysis of testis lysates from heterozygous and homozygous *Fam209^−3^* or *Fam20^+1^* mice, using antibody against DPY19L2, showing that DPY19L2 is present in all. Actin was used as a loading control. −3 Het, *Fam209^+/−3^* mice; −3/−3, *Fam209^−3/−3^* mice; +1 Het, *Fam209^+/+1^* mice; +1 KO, *Fam209^+1/+1^* mice.

**Fig. 6.**
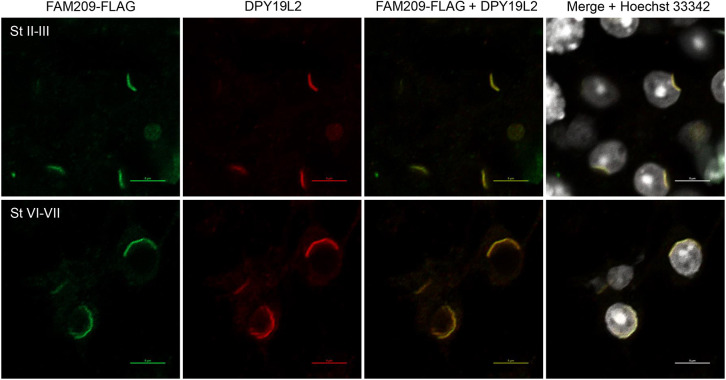
**FAM209 colocalizes with DPY19L2.** Immunostaining of *Fam209*^*Flag/Flag*^ testis. Staining with anti-FLAG antibody shows that FAM209-FLAG colocalizes with the inner nuclear membrane protein DPY19L2 in developing spermatids. Shown are stage II–III and stage VI–VII spermatids. Scale bars: 5 µm.

Taking advantage of the endogenously tagged *Fam209*-FLAG mice, we wanted to determine the proteome of FAM209 by using immunoprecipitation (IP), to begin to elucidate its role in acrosome biogenesis. To increase isolation of acrosomal and nuclear membranes, CHAPS detergent was used at 1% during lysis of tissue. Also, to minimize non-specific isolation of proteins, FLAG peptide under non-denaturing conditions (i.e. a neutral pH) was used to elute protein complexes from anti-FLAG resin (Fig. S5A). FAM209-FLAG protein complexes were isolated from testis lysates of *Fam209^Flag/Flag^* mice, with wild-type mice lacking the FLAG epitope serving as a control (Fig. 5A). Both samples were submitted to mass spectrometry (MS) analysis. Three independent IP experiments identified calmegin (CLGN), nesprin-2 (SYNE2), seipin (BSCL2), nuclear pore membrane glycoprotein 210-like (NUP210L) and DPY19L2 to be associated with FAM209-FLAG (Fig. 5B, Tables S1-S3). CLGN, an endoplasmic reticulum (ER) chaperone required for transmembrane protein folding, was confirmed to be associated with FAM209 by western blot analysis (Fig. S5B) (Ikawa et al., 1997). SYNE2 is part of a family of proteins important in sperm-head formation (Apel et al., 2000; Göb et al., 2010; Zhang et al., 2005). BSCL2 is an ER transmembrane protein implicated in acrosome formation (El Zowalaty et al., 2015; Magré et al., 2001). NUP210L is a testis-specific NUP210 paralog implicated in acrosome formation (Arafah et al., 2021; Walters et al., 2009). Probable C-mannosyltransferase DPY19L2 has been implicated in globozoospermia in infertile men (spermatogenic failure type 9) and localizes to the inner nuclear envelope in the region of the developing acrosome (Koscinski et al., 2011; Pierre et al., 2012). Deletion of *Dpy19l2* in mice leads to globozoospermia and acrosome loss (Pierre et al., 2012). We obtained a polyclonal antibody against mouse DPY19L2, and western blot analysis confirmed that FAM209 and DPY19L2 interact (Fig. 5C). To see whether absence of *Fam209* affects expression of *Dpy19l2*, we examined DPY19L2 protein levels in testis lysates of *Fam209* mutants. Western blot analysis showed no changes of DPY19L2 levels in the different *Fam209* mutants (Fig. 5D). Immunofluorescence (IF) staining of DPY19L2 and FLAG in *Fam209^Flag/Flag^* testes demonstrated colocalization of FAM209 and DPY19L2 to the inner nuclear envelope (Fig. 6, Movies 3 and 4). To determine whether FAM209 affects DPY19L2 localization, we performed IF staining of DPY19L2 in *Fam209^−3/−3^* and *Fam209^+1/+1^* testis cryosections. IF staining showed DPY19L2 in *Fam209^−3/−3^* and *Fam209^+1/+1^* round spermatids (Fig. S6A and B), which persisted during spermiogenesis in *Fam209^−3/−3^* but was lost in *Fam209^+1/+1^* testes as spermatids enter the elongation phase. These results suggested that DPY19L2 does not depend on FAM209 for localization but, potentially, for stabilization during the later stages of acrosome biogenesis. Taken together, these data show that FAM209 associates with DPY19L2 in the inner nuclear envelope and both proteins are crucial for acrosome biogenesis.

### FAM209 affects the proteome of DPY19L2

**Fig. 7.**
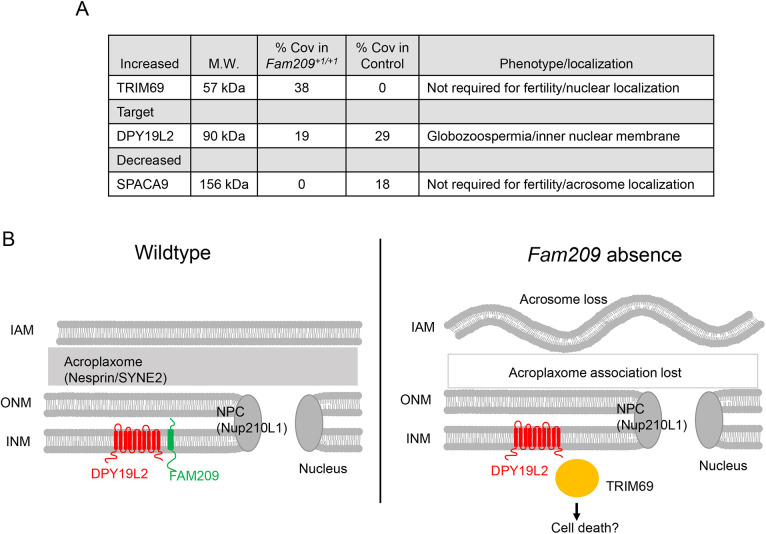
**FAM209 affects proteins associated with DPY19L2.** (A) Mass spectrometry analysis data, showing proteins identified to associate with DPY19L2 in the presence or absence of FAM209 (% Cov, percent coverage). (B) Schematic of how FAM209 might affect the function of DPY19L2. In the absence of FAM209 (right), DPY19L2 inappropriately binds to other proteins, such as TRIM69, leading to loss of the acrosome. IAM, inner acrosome membrane; ONM, outer nuclear membrane; INM, inner nuclear membrane; NPC, nuclear pore complex

As IP and MS analyses consistently showed DPY19L2 associated with FAM209, we, therefore, assumed that the two proteins are in a protein complex. We also reasoned that absence of FAM209 might affect the composition of a potential DPY19L2 complex. To test this, we used IP to pull down DPY19L2 from testis lysates in the presence or absence of FAM209, as well as MS to identify any associated proteins. We used testis lysates from *Fam209^+1/+1^* mice because western blotting confirmed the total absence of FAM209 in those mice. We successfully used IP and MS to identify the target protein DPY19L2 (Fig. 7A, Table S4); among the proteins that showed decreased association with DPY19L2 in the absence of FAM209 is the acrosome protein SPACA9 (Bhattacharya et al., 2013). Fortuitously, our group was able to generate *Spaca9* KO mice using CRISPR/Cas9 (Fig. S7) and to show that SPACA9 is not required for male fertility. Our data show that in the absence of FAM209, association of DPY19L2 with the testis-enriched nuclear E3 ubiquitin ligase TRIM69 (also known as RNF36) is increased (Fig. 7A). TRIM69 has been shown to induce apoptosis when overexpressed (Shyu et al., 2003) but also that it protects against apoptosis in other contexts (Li et al., 2019). Together, these data demonstrate that FAM209 affects proteins associated with DPY19L2, and this might lead to defects in acrosome development and spermiogenesis (Fig. 7B).

## DISCUSSION

We have shown that *Fam209* is an evolutionarily conserved, testis-specific gene that is only present in mammals; expression of this gene was detected at RNA level at postnatal day 20 (P20). At P20, the leading edge of spermatogenesis enters the haploid phase to produce round spermatids. Mouse FAM209 has been predicted to be 170 aa long, with the first 20 aa being a signal peptide that is cleaved, and a transmembrane domain between aa positions 56 and 74 (Käll et al., 2007). Based on topology prediction software, it was predicted that the N-terminus of FAM209 is luminal/extracellular and the C-terminus cytoplasmic/nucleoplasmic. FAM209 orthologues have no homology to other proteins, have no recognizable functional domains and are annotated in the NCBI database as containing a domain of unknown function specific to FAM209 (the FAM209 domain; PFAM domain PF15206). The primate lineage appears to have undergone gene duplication of *FAM209*, such that humans contain *FAM209A* and *FAM209B* (Fagerberg et al., 2014). However, the functional significance of gene duplication in human is unclear as the two paralogues are 88% identical and expression of both genes is increased in testis. However, based on our cell expression data and the single cysteine within the N-terminal domain, i.e. the region that is in the perinuclear space and would have been inside the endoplasmic reticulum, it is possible that these paralogs are disulfide-linked. About 200 coding variants of *FAM209A/B* have been listed in the dbSNP database; however, no clinical significance has been assigned to either. It is possible that, due to the gene duplication of FAM209, single deleterious mutations of either gene do not present with a clinical phenotype unless associated with functional heterodimerization of FAM209A and FAM209B. However, as far as we are aware, mutations of *FAM209* have not been screened in infertile men.

To determine the function of FAM209 during spermatogenesis, we used the CRISPR/Cas9 system to generate *Fam209* mutants in mouse. We generated an in-frame deletion mutant that lacks three thymine nucleotides – resulting in lack of phenylalanine 43, and a +1-frameshift insertion mutant featuring an additional thymine nucleotide at position 129 of the coding sequence. At protein level, FAM209 is still present in testes of −3 mutant males but absent in those of +1 mutant males. This difference in protein expression between the two alleles might be due to nonsense-mediated decay of the +1 transcript and read-through of the −3 allele. When examining spermatogenesis by using histology and electron microscopy, and through immunofluorescence analysis using spermatogenesis markers, the −3 allele shows fewer defects in spermatogenesis compared to the +1 allele. Based on these data, the −3 allele is a hypomorph allele, whereas the +1 allele is an amorph allele. Breeding studies further support that the −3 allele is a hypomorph, as one out of five −3 homozygous males was able to sire two pups over two litters, whereas all +1 homozygous males were sterile. As mentioned earlier, the transcript product from the +1 allele probably undergoes nonsense-mediated decay that would explain the severity of phenotype. However, the milder phenotype of the −3 allele might be due to reduced levels of FAM209 protein or deletion of the phenylalanine (F) at position 43. F43 becomes position 23 after the signal peptide of FAM209 is cleaved and is 13 aa upstream from the transmembrane domain. As phenylalanine has several roles in protein function and structure (Betts and Russell, 2003), F43 might stabilize or be important for FAM209 function. The −3 allele does not appear to be dominant negative since no observable defect was present in heterozygous males.

To further examine the FAM209 mechanism of action, we used the *Fam209^Flag/Flag^* mouse because FAM209 antibodies are unsuitable for IF and because we were concerned about the non-specific bands detected by western blot analysis. The *Fam209^Flag/Flag^* knock-in mouse allowed us to localize FAM209 to a region beneath the acrosome, specifically to the inner nuclear membrane. No nuclear localization signal was detected in FAM209; however, inner nuclear membrane localization is distinct from nuclear trafficking of cyto- and nucleoplasmic proteins (Katta et al., 2014). Our localization study confirmed the proteomic analysis of human sperm nuclei, which detected both FAM209A and FAM209B (de Mateo et al., 2011; Ficarro et al., 2003). Examination of mouse FAM209-associated proteins detected DPY19L2, a protein that contains ten transmembrane domains, is localized to the inner nuclear envelope and was first found to be implicated in globozoospermia of infertile men ∼10 years ago (Harbuz et al., 2011; Koscinski et al., 2011; Pierre et al., 2012). The mouse *Dpy19l2* KO was published the following year (Pierre et al., 2012); however, since then, the proteome of DPY19L2 has remained elusive – probably owing to the difficulties of working with transmembrane proteins. DPY19L2 is an orthologue of *Caenorhabditis elegans* DPY-19 that encodes a C-mannosyltransferase (Brenner, 1974; Buettner et al., 2013) and facilitates addition of mannose to the indole ring of tryptophan (W) residues, the consensus sequence being WxxW – with x representing any amino acid (Julenius, 2007; Krieg et al., 1998; Löffler et al., 1996). FAM209 has several W residues but does not contain this consensus sequence. In addition, DPY19L2 has not been shown to comprise C-mannosylation activity; so, whether FAM209 is modified by DPY19L2 remains an open question. The most relevant finding is that FAM209 is the first protein discovered to associate with DPY19L2 *in vivo*, which allowed us to determine whether FAM209 affects DPY19L2 function by examining the associated proteins of DPY19L2. We attempted to determine the proteome of DPY19l2 by using IP and MS analyses in the presence and the absence of FAM209. IP and MS analyses showed increased association of DPY19L2 with TRIM69, an E3 ubiquitin ligase localized to the nucleus of spermatids. *Trim69* has been shown to be non-essential for fertility (He et al., 2021; Shyu et al., 2001); however, TRIM69 has been shown to induce or protect against apoptosis (Li et al., 2019; Shyu et al., 2003). It is possible that aberrant association of DPY19L2 with TRIM69 leads to inappropriate ubiquitylation of other proteins associated with DPY19L2, which then causes the loss of acrosome phenotype. Another finding of IP and MS analyses was the decreased association of DPY19L2 with the acrosome protein SPACA9 (Bhattacharya et al., 2013). However, deleting the majority of the *Spaca9* coding sequence, showed no defect regarding the fertility of male mice. This suggests that loss of the DPY19L2-SPACA9 association is not the cause of the acrosome-less phenotype seen in the *Fam209^+1^* allele. We should emphasize that our protocol regarding IP of DPY19L2 was not as successful in decreasing signal from mouse IgG heavy and light chain as compared to the IP of FAM209-FLAG. A tagged version of DPY19L2 seems to be essential to determine the function of the FAM209–DPY19L2 complex and how it assists acrosome biogenesis.

## MATERIALS AND METHODS

### Animal models and ethics statement

*Fam209*^+1^ or *Fam209*^−3^ mice were obtained by pronuclear injection of a CRISPR/Cas9 RNP particle targeting the first exon of *Fam209* (Table S5). *Fam209^FLAG^* mice were generated by injecting pX330 expression plasmid that contains a single-guide RNA targeting the stop codon as well as a 130 DNA oligomer containing the FLAG sequence and short homology arms into egg cells (Table S5). *Spaca9*^−3977^ mice were generated by electroporation of CRISPR/Cas9 RNP particles and two guide RNAs to delete the majority of the *Spaca9* coding sequence into zygotes (Table S5). All mice were purchased from CLEA Japan (Tokyo, Japan) or Japan SLC (Shizuoka, Japan) and maintained on a B6D2F1 background. All mice were housed in specific pathogen-free animal facilities with a light:dark cycle of 12h:12h.

All animals used in this study were approved by the Institutional Animal Care and Use Committees of the Research Institute for Microbial Diseases of Osaka University (Osaka, Japan). All strains used in this study (*Fam209*^+1^, *Fam209*^−3^, *Fam209^FLAG^*, *Spaca9^−3977^*) will be deposited as bioresources at CARD (Kumamoto University) and RIKEN and be available to researchers. The strains deposited are as follows: B6D2-Fam209^em1Osb^ for *Fam209*^−3^ mice; B6D2-Fam209^em2Osb^ for *Fam209*^+1^ mice; B6D2-Fam209^em3(Fam209/FLAG)Osb^ for *Fam209*^*FLAG*^ mice; B6D2-Spaca9^em1Osb^ for *Spaca*^−3977^.

### RT-PCR

Mouse cDNA was obtained from multiple tissues of B6D2F1 and C57CL6/129SvEv hybrid adult male and female mice, and from testes of male mice aged between 5 and 60 days (Miyata et al., 2016). Primers and amplification conditions for each gene are summarized in Table S5.

### Sequence comparisons

Sequence comparisons between FAM209 homologs were done using BLAST or Clustal Omega (Madeira et al., 2019). FAM209 sequences of platypus, opossum, rat, bovine, mouse and human were included.

### Mating tests

Three wild-type B6D2F1, three heterozygous *Fam209^+/−3^* or *Fam209^+/+1^*, three homozygous *Fam209^+1/+1^* and five homozygous *Fam209^−3/−3^* sexually mature males were paired with two to three 6-week-old wild-type females for 8 weeks. Copulatory plug formation was confirmed and the number of pups per litter was recorded for all genotypes.

### Western blot analysis of mouse testes

Testes dissected from wild-type, heterozygous *Fam209^−3/−3^* or *Fam209^+1/+^* were lysed in 1 ml CHAPS buffer (50 mM Tris pH 7.5, 150 mM NaCl, 1 mM DTT, 1% CHAPS, protease and phosphatase inhibitors). Lysates were clarified during a 10 min spin (18,000 ***g*** at 4°C). Care was used to include the upper lipid layer (white fraction) to the supernatant. The protein concentration was determined using the Bradford method; 50 µg per sample were loaded per well. Western blot analysis was performed using the TransBlot Turbo transfer system (BioRad). PVDF membranes were blocked with TBS supplemented with 0.05% Tween and 5% milk, and washed with TBS with 0.05% Tween and 0.5% milk. Both primary and secondary antibodies were diluted in washing buffer. Chemi-Lumi One Super (catalog no. 02230-30; Nacalai, Kyoto, Japan) was used for chemiluminescence and detected using Image Quant. Antibodies used were rabbit polyclonal anti-FLAG (MBL; catalog no. PM020) at 1:1000; rabbit polyclonal anti-DPY19L2 at 1:1000 (Pierre et al., 2012); rabbit anti-CLGN at 1:1000 (Ikawa et al., 1997); and goat polyclonal anti-actin at 1:1000 (Santa Cruz, catalog no. sc1616).

### FAM209 antibody production

An antibody production service (Sigma) was utilized to produce our FAM209 antibody. The peptide sequence LKFRGDGENKEQHPPGLRG, corresponding to aa 74–92 of mouse FAM209, was used to immunize one rabbit. After 54 days, the blood serum was extracted and anti-FAM209 antibodies were purified using affinity resin (Thermo Sulfo-Link) generated with the immunizing peptide.

### Histology

For Periodic acid–Schiff (PAS) staining, adult testes (obtained from mice >2 months) were fixed in Bouin's fixative, processed and embedded in paraffin. Sections were cut at 5 μm thickness. Sections were then deparaffinized, rehydrated, stained with PAS reagent, counterstained with hematoxylin, dehydrated and mounted with Permount. Images were acquired using an Olympus BX53 upright microscope with an Olympus DP74 camera.

### Sperm analysis

Sperm motility was analyzed as previously described (Miyata et al., 2015). Sperm from *Fam209^+/−3^*, *Fam209^−3/−3^*, *Fam209^+1/+1^* or *Fam209^+1/+1^* mice were extracted into TYH medium (Toyoda and Yokoyama, 2016) and incubated at 37°C under 5% CO2. Computer-assisted sperm analysis (CASA) (Hamilton Thorne) with CEROS II software was used to measure sperm motility after incubation for 10 min and 120 min. At least three males were used for each genotype.

### Immunofluorescence

For testis cryosections, immunostaining was performed as previously described (Castañeda et al., 2014). Primary antibodies were rat anti-IZUMO1 1:200 (Inoue et al., 2005); rabbit anti-SPACA1 1:500 (Fujihara et al., 2012); rabbit anti-DPY19L2 1:1000 (Pierre et al., 2012); rabbit anti-FLAG 1:500 (MBL catalog no. PM020); and mouse anti-FLAG 1:200 (Sigma; M2). Secondary antibodies were Alexa-Fluor-488 anti-rabbit, Alexa-Fluor-546 anti-mouse, Alexa-Fluor-546 anti-rat – all used at 1:1000. Images were acquired using a Nikon Eclipse Ti confocal microscope followed by deconvolution with Nikon Imaging Software.

### Immunoelectron microscopy

Immunoelectron microscopy (immuno-EM) was performed as described by Shimada et al. (2021).Testes were obtained from anesthetised wild-type and *Fam209^FLAG^* adult males. Testes were perfusion fixed, cut into 2 mm pieces and post-fixed with 4% PFA in 100 mM phosphate buffer pH 7.4. Tissue pieces were incubated in increasing concentrations of sucrose (4%, 10%, 15%, 20%) in 100 mM phosphate buffer, embedded in optimal cutting temperature compound (Tissue Tek, catalog no. 4583), and snap-frozen in liquid nitrogen. Cryosections were cut at 6 µm thickness and placed on MAS-GP adhesion microscope slides (Matsunami Glass) and air dried for 30 min. The samples were permeabilized with 0.25% saponin in 100 mM phosphate buffer for 30 min and blocked with blocking solution (100 mM phosphate buffer containing 0.01% saponin, 10% BSA, 10% normal goat serum and 0.1% cold-water fish skin gelatin) for 30 min. The samples were incubated with anti-FLAG (MBL, catalog no. PM020) diluted 1:300 in blocking buffer overnight at 4°C, washed with 100 mM phosphate buffer supplemented with 0.005% saponin and incubated with 1.4 nm labeled goat anti-rabbit IgG 1:400 (Nanogold, Nanoprobes, Yaphank, NY) in blocking solution for 2 h. The samples were then washed once and fixed with 1% glutaraldehyde in 100 mM phosphate buffer for 10 min. Sections were washed in PBS containing 50 mM glycine, followed by washing in PBS containing 1% BSA. Gold labeling was intensified using the GoldEnhance EM kit (Nanoprobes) for 3 min. The gold intensification solution was removed, and sections were soaked in 1% sodium thiosulfate solution for a few seconds and washed once in H_2_O. The sections were post-fixed in 1% OsO_4_ and 1.5% K_4_[Fe(CN)_6_] in 100 mM phosphate buffer for 1 h. Samples were dehydrated in a graded series of ethanol and embedded in epoxy resin. Ultra-thin sections (80 nm thick) were stained with 8% uranyl acetate and lead staining solution. The samples were examined using a JEM-1400 plus electron microscope (JEOL, Tokyo, Japan) at 80 kV with a CCD Veleta 2 K×2 K camera (Olympus).

### Immunoprecipitation using testis tissue

Anti-FLAG affinity resin (Sigma, catalog no. A2220) was used for affinity purification of FAM209-FLAG. Testes from three wild-type or *Fam209^FLAG^* adults aged 2 months or older were dissected and lysed in 2 ml CHAPS buffer (see above). Lysates were clarified during a 10 min spin (18,000 ***g*** at 4°C). Care was used to include the upper lipid layer (white fraction) with the supernatant. 500 μl of anti-FLAG M2 resin was washed with 10 volumes of CHAPS buffer and incubated with clarified lysate for 1 h at 4°C with gentle mixing. The resin was then washed again with 20 volumes of CHAPS buffer. FAM209 protein complexes were then eluted with FLAG peptide (200 μg/ml diluted in CHAPS buffer) and samples were analyzed using the BIKEN Mass Spec core facility at Osaka University.

For IP of DPY19L2, ∼15 µg of purified anti-DPY19L2 (Pierre et al., 2012) was conjugated to Protein A/G resin using the Co-IP kit (catalogue no.: 26147; Thermo Scientific) to generate anti-DPY19L2 resin. Testes from one *Fam209^FLAG^* adult (aged 2 months or older) or one *Fam209^+1/+1^* adult were dissected and lysed in 500 µl CHAPS buffer (see above). Lysates were clarified during a 10-min spin (18,000 ***g*** at 4°C). Care was used to include the upper lipid layer (white fraction) with the supernatant. The lysates were then incubated with anti-DPY19L2 resin for 3 h at 4°C. Resin was washed 5× with CHAPS buffer and proteins were eluted off the beads using Pierce IgG Elution Buffer (Thermo Scientific, catalog no. 21004). The samples were analysed by using the BIKEN Mass Spec core facility at Osaka University.

### Statistical analysis

No power analysis was used to determine sample size. Statistical analysis was done when using three or more males for experiments. Fisher's exact test or Student's *t*-test were used to examine statistical significance. *P*-values between 0.05 and 0.001 were considered significant (*), whereas *P*<0.001 was considered highly significant (**).

## Supplementary Material

Supplementary information
