# Supplementary material for: FAM209 associates with DPY19L2, and is required for sperm acrosome biogenesis and fertility in mice
Source: J Cell Sci. 2021 Nov 1;134(21):jcs259206. doi: 10.1242/jcs.259206 (PMC8627553; doi:10.1242/jcs.259206)
Supplement: Supplementary information [file joces-134-259206-s1.pdf]

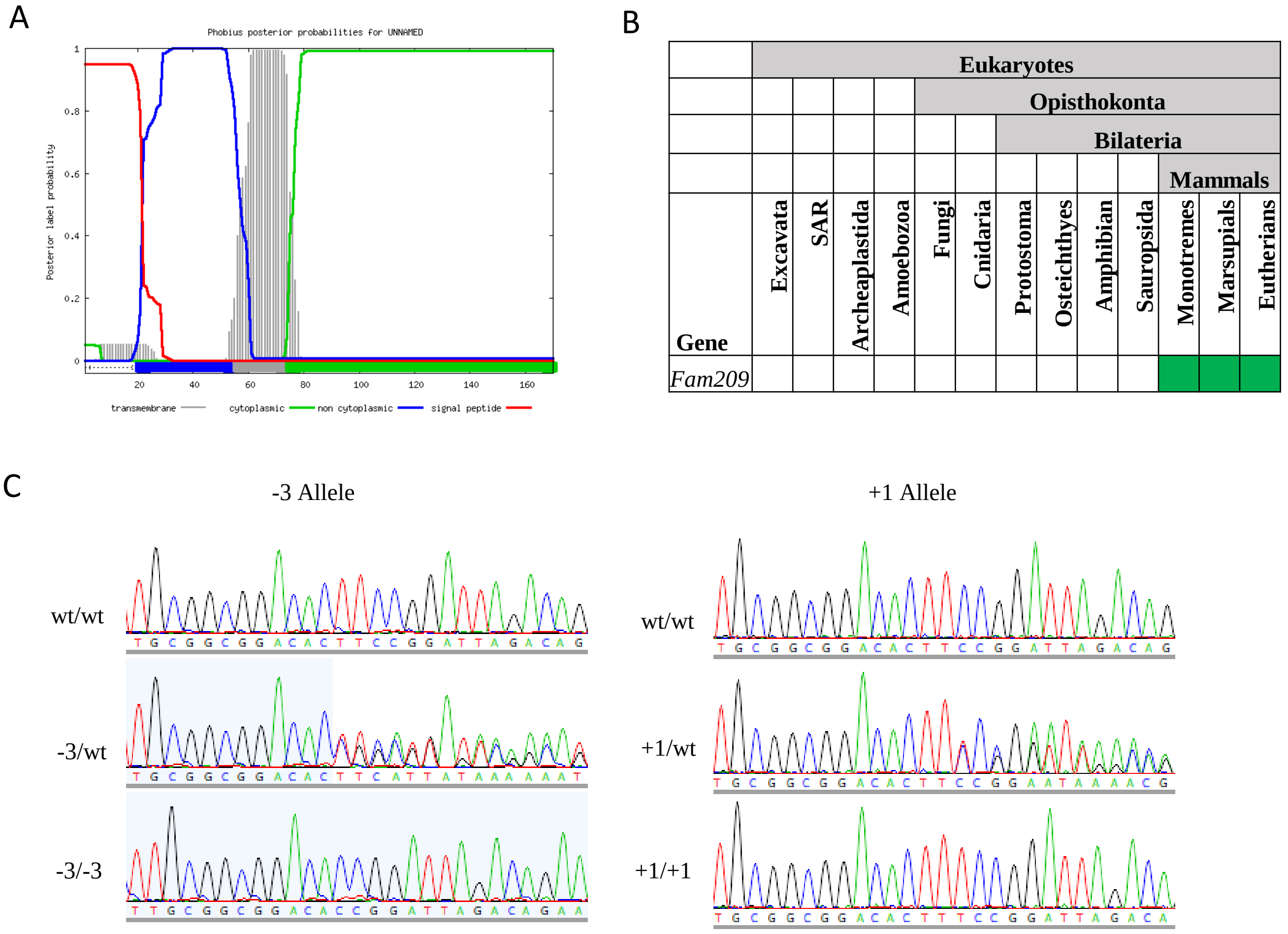

Fig. S1. FAM209 is an evolutionary conserved transmembrane protein. A Plot of FAM209 analysis using Phobius transmembrane and signal peptide analysis (Stockholm Bioinformatics Center). **B** Chart showing the presence of *Fam209* in various eukaryotic lineages. **C** DNA chromatograms distinguishing between the -3 and +1 *Fam209* alleles.

Journal of Cell Science • Supplementary information

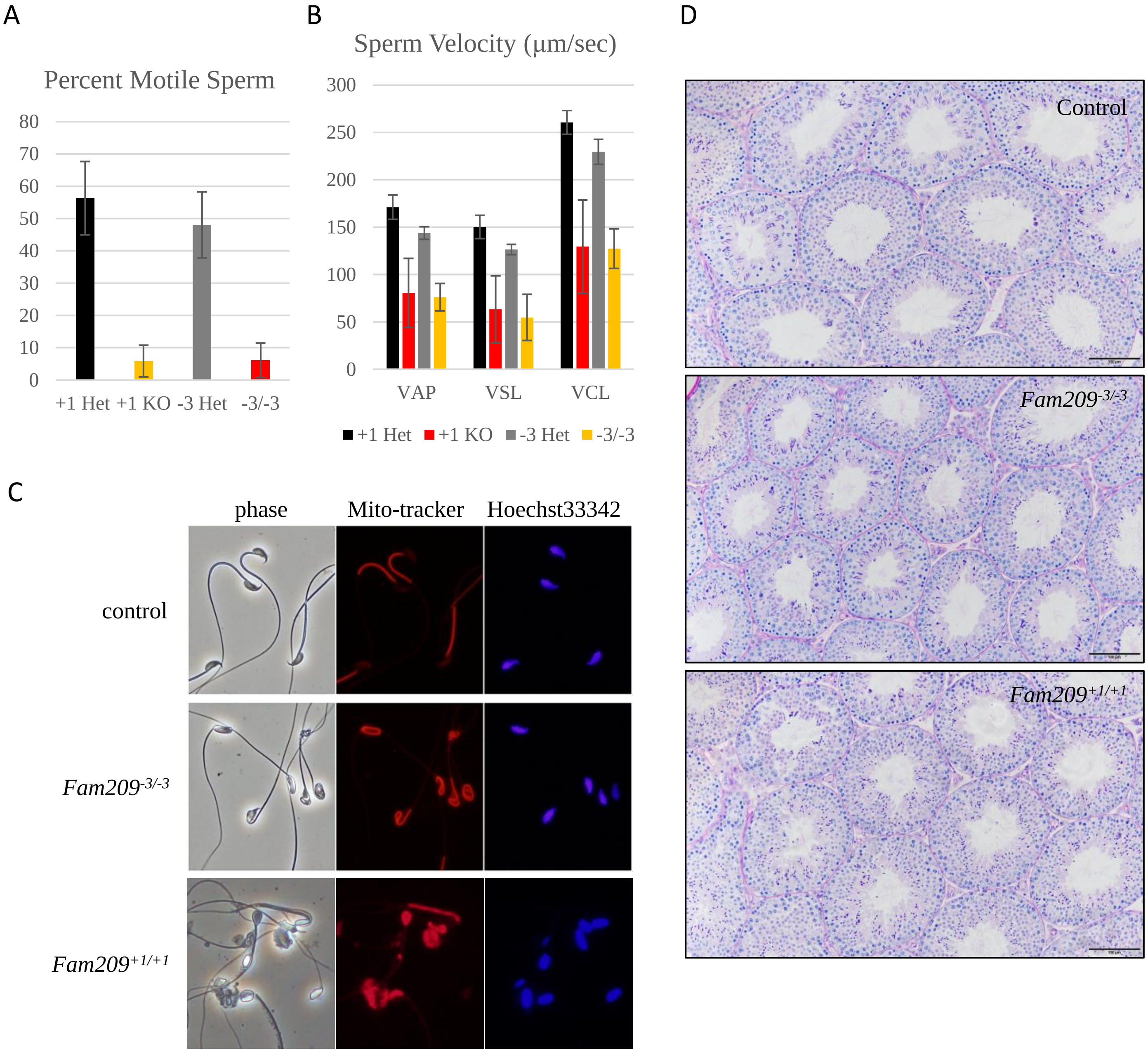

Fig. S2. *Fam209*$^{+1/+1}$ mice display a stronger phenotype than *Fam209*$^{-3/-3}$ mutant mice. A Measurements of the percent of motile sperm isolated from the cauda epididymis from *Fam209*$^{wt/-3}$, *Fam209*$^{-3/-3}$, *Fam209*$^{wt/+1}$, and *Fam209*$^{+1/+1}$ mice (n≥3 for each genotype). **B** The velocity parameters of sperm as determined by CASA for each genotype (n≥3 for each genotype). **C** Morphology of sperm from control, *Fam209*$^{-3/-3}$, and *Fam209*$^{+1/+1}$ mice. **D** PAS-stained testis cross sections from control, *Fam209*$^{-3/-3}$, *Fam209*$^{+1/+1}$ mice. *Fam209*$^{-3/-3}$ histology is grossly comparable to control. Abnormally condensed elongating spermatids are detected in *Fam209*$^{+1/+1}$ mice. Scale bars = 100 µm.

Journal of Cell Science • Supplementary information

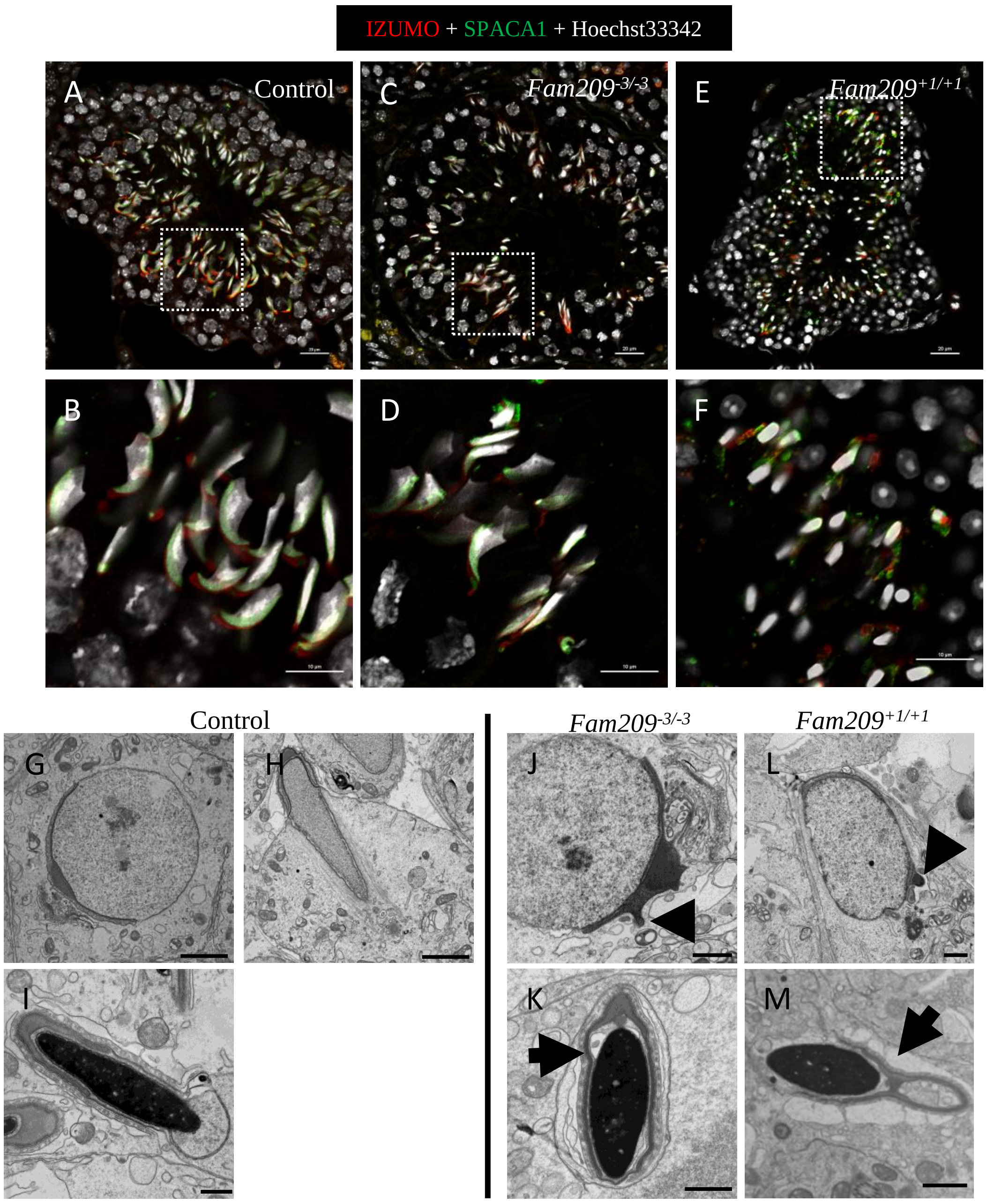

Fig. S3. Acrosome components mislocalize in *Fam209* mutant spermatids. A IZUMO1 and SPACA1, two acrosome membrane proteins, localize to the acrosome in elongating spermatids. Scale bars = 20 µm. **B** Close up of figure **A**. Scale bars = 10 µm. **C** IZUMO1 and SPACA1 show slight mislocalization in *Fam209*$^{-3/-3}$ spermatids. Scale bars = 20 µm. **D** Close up of figure **C**. Scale bars = 10 µm. **E** IZUMO1 and SPACA1 shows severe mislocalization in *Fam209*$^{+1/+1}$ spermatids. Scale bars = 20 µm. **F** Close up of figure **E**. Scale bars = 10 µm. **G** Round spermatid in the cap phase of acrosome development. Scale bar = 2 µm. **H** Spermatid in the acrosome phase of acrosome development. Scale bar = 2 µm. **I** Spermatid in the in the maturation phase of acrosome development. Scale bar = 0.5 µm. **J** *Fam209*$^{-3/-3}$ spermatid in the cap phase detects abnormal lobes in the acrosome cap (arrowhead). Scale bars = 1 µm. **K** *Fam209*$^{-3/-3}$ spermatid in the maturation phase shows loose association of the acrosome with the nucleus (arrows). Scale bar = 1 µm. **L** *Fam209*$^{+1/+1}$ spermatid in the acrosome phase detects abnormal organization of the acrosome granule at step 9. Scale bar = 1 µm. **M** *Fam209*$^{+1/+1}$ spermatid in the maturation phase shows loss of the acrosome in elongated spermatids (arrow). Scale bar = 1 µm.

Journal of Cell Science • Supplementary information

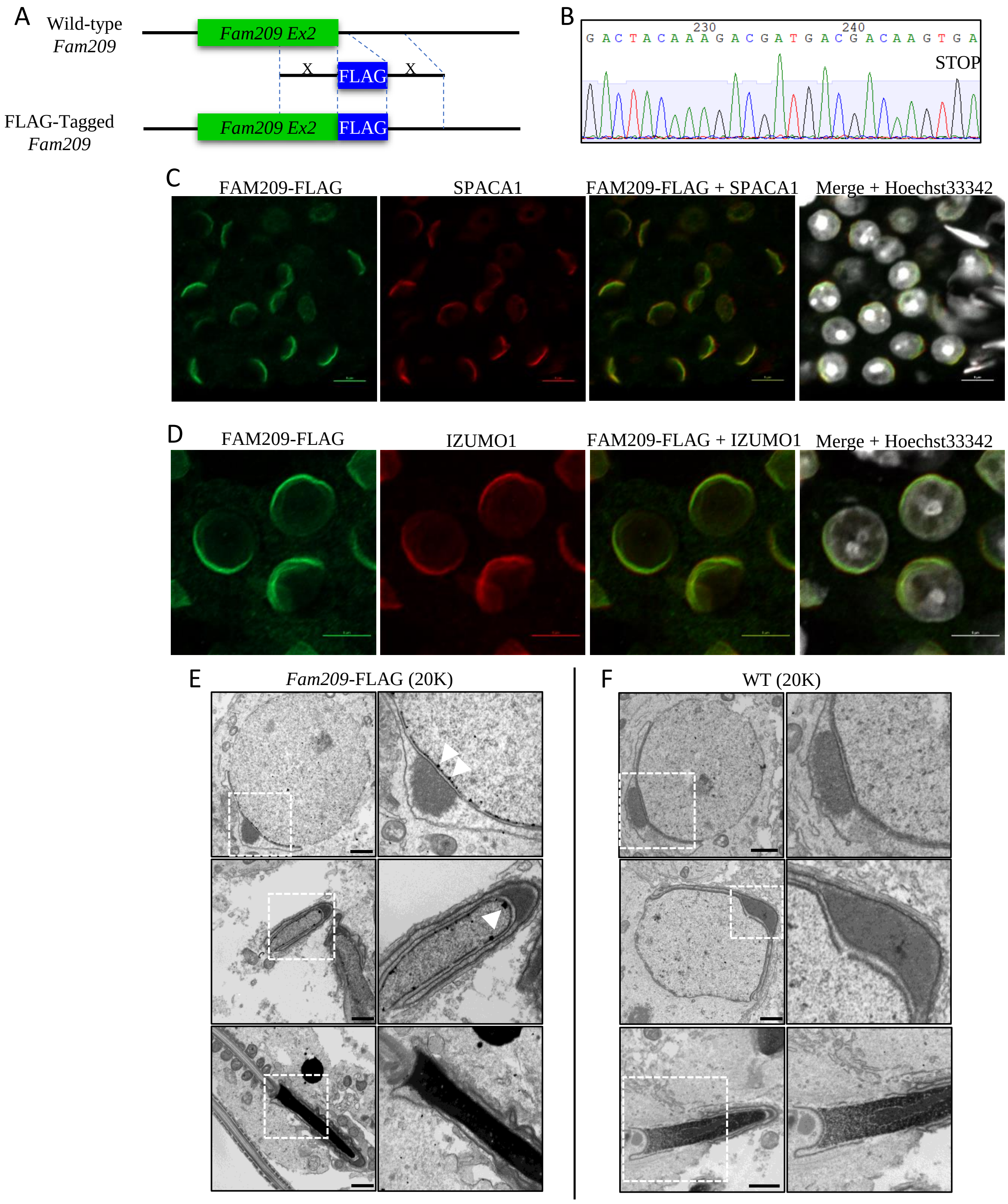

Fig. S4. FAM209 colocalizes beneath acrosome components. A Schematic showing how *Fam209*-FLAG mice were generated. **B** Chromatogram of *Fam209*-FLAG genomic sequencing. **C** Localization of FAM209-FLAG with anti-FLAG immunostaining (green signal) localizes underneath SPACA1 (red signal), an inner acrosomal membrane protein. Scale bars = 5 µm. **D** Localization of FAM209-FLAG with anti-FLAG immunostaining (green signal) localizes underneath IZUMO1 (red signal), an acrosomal membrane protein. Scale bars = 5 µm. **E** Immunogold labeling against FLAG in *Fam209*$^{FLAG/FLAG}$ ultra-thin testis sections. Arrows highlight gold particles localizing to the inner nuclear membrane in spermatids. Gold particles are not detected near the nuclear envelope in Step 15 spermatids. Scale bars = 1 µm. **F** Immunogold labeling against FLAG in wild-type ultra-thin testis sections. Gold particles are not detected near the inner nuclear envelop in any spermatid step. Scale bars = 1 µm.

Journal of Cell Science • Supplementary information

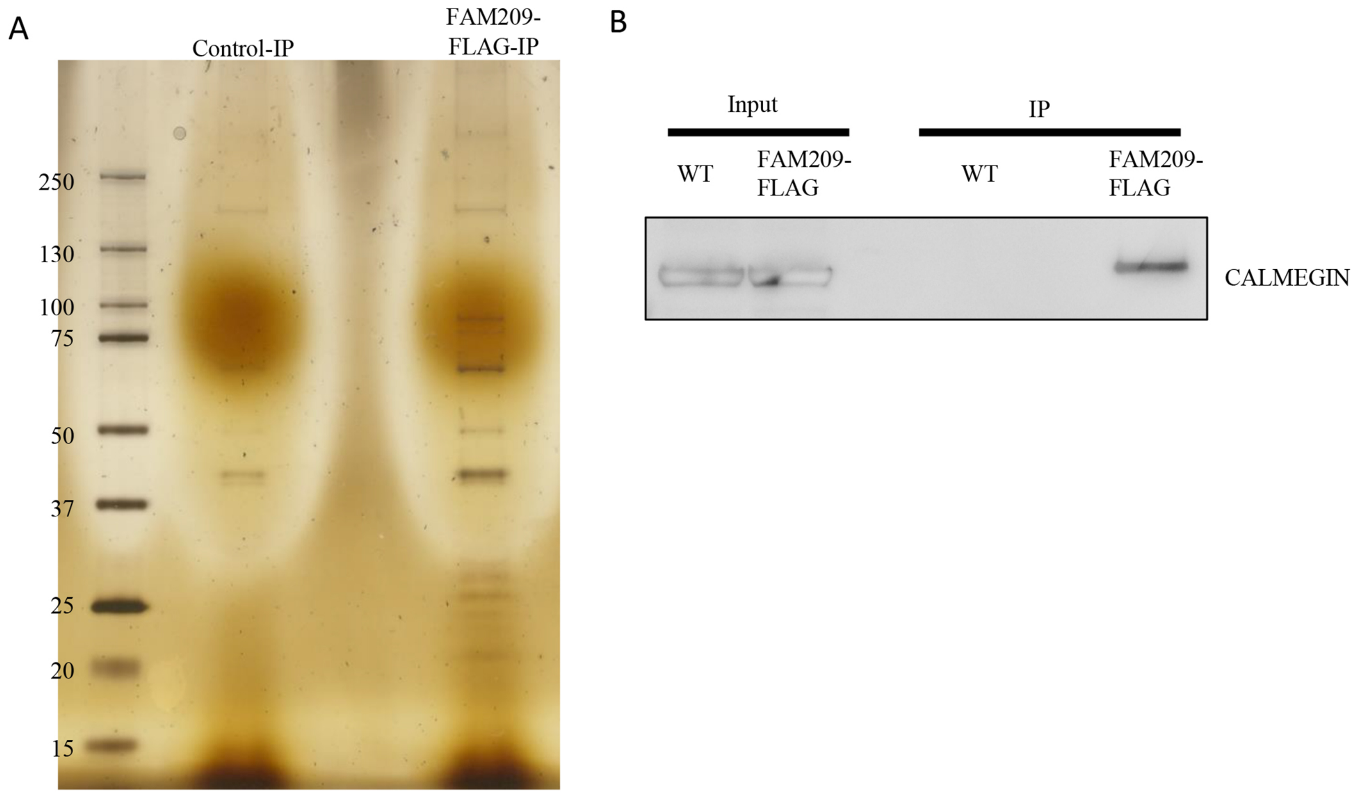

Fig. S5. Immunoprecipitation of FAM209. A Silver staining identifies several bands specific to the FAM209 immunoprecipitation absent in the control (genotype = wild type). **B** Western blot analysis of FAM209-FLAG immunoprecipitation identifies CALMEGIN, an ER chaperone, as co-immunoprecipitating with FAM209-FLAG.

Journal of Cell Science • Supplementary information

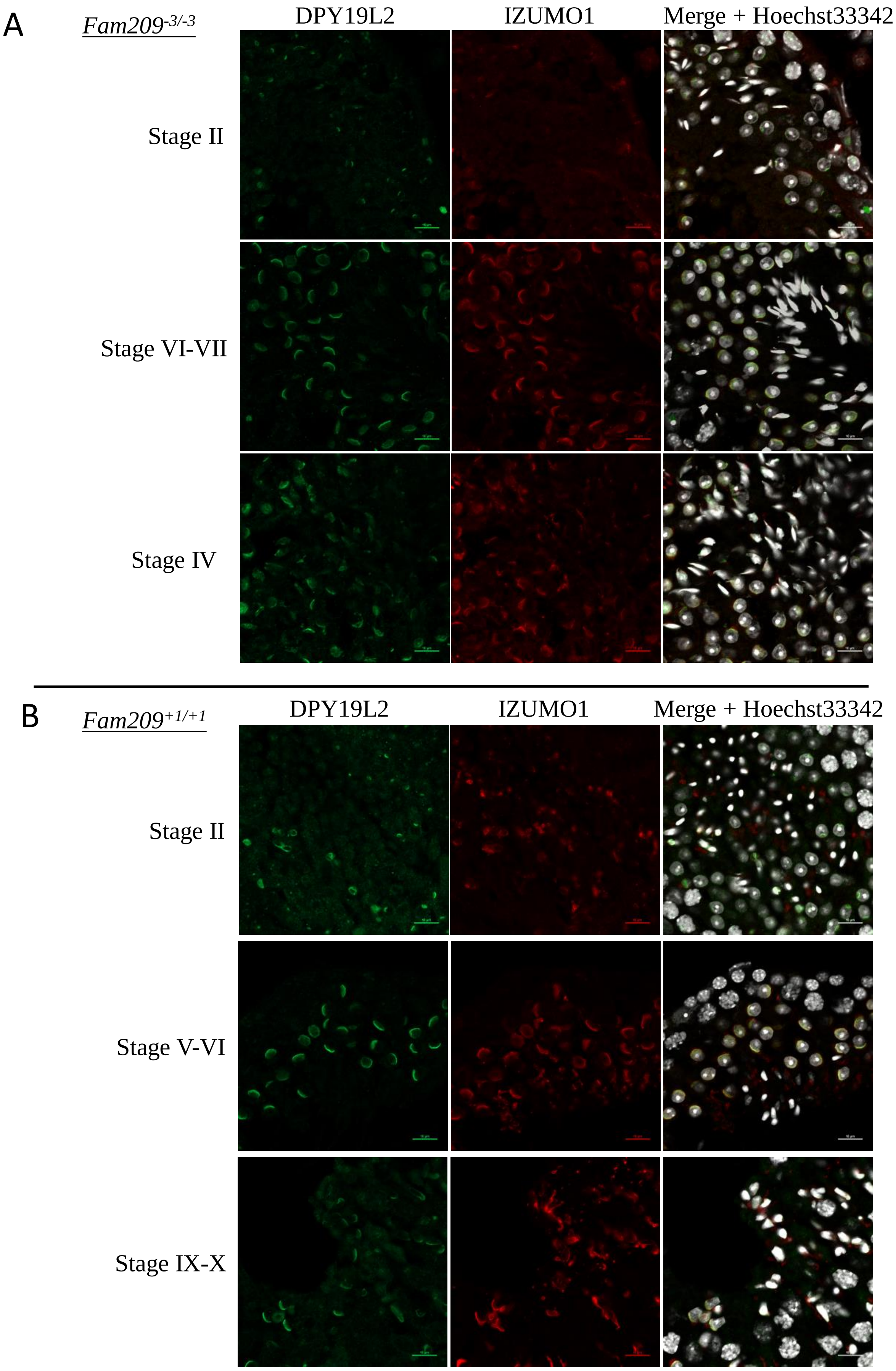

Fig. S6. DPY19L2 localization is not affected in *Fam209*$^{-3/-3}$ spermatids or in *Fam209*$^{+1/+1}$ early spermatids. A DPY19L2 and IZUMO1 immuno-localization at various stages of spermatogenesis in *Fam209*$^{-3/-3}$. Scale bars = 10 µm. **B** DPY19L2 and IZUMO1 immuno-localization at various stages of spermatogenesis. DPY19L2 signal persists in *Fam209*$^{+1/+1}$ round spermatids and decreases as spermatids enter the elongation stage early spermatids**.** Scale bars = 10 µm.

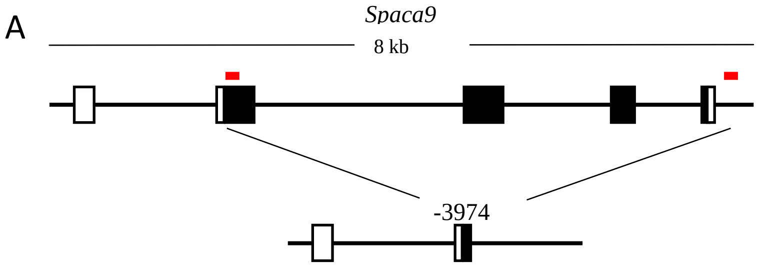

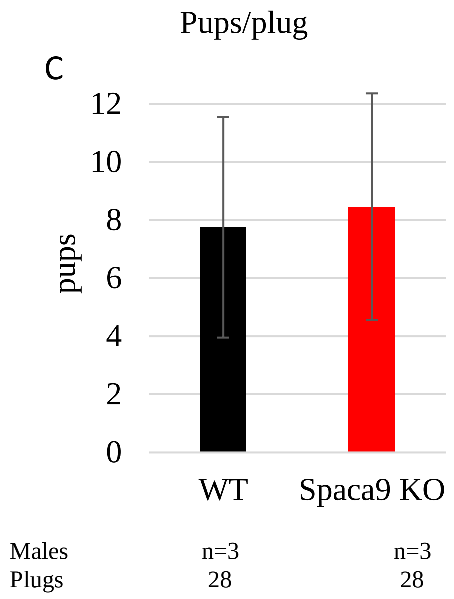

B

gRNA	Target	PAM
#1	CTTCTGCTCGATGCTGCGGA	GGG
#2	GATTTTGTCGGGTAAATCCC	AGG

Fig. S7. *Spaca9* is not required for male fertility. A Schematic of the *Spaca9* locus and the targeted deletion. **B** Guide RNAs used to generate the *Spaca9* deletion. **C** Fertility analysis of *Spaca9* KO males.

Journal of Cell Science • Supplementary information

Table S1. Mass spectrometry results of the first FAM209-FLAG immunoprecipitation (IP). IP values from Control (wildtype) and *Fam209*$^{Flag/Flag}$ are presented as normalized total spectra.

Click here to download Table S1

Table S2. Mass spectrometry results of the second FAM209-FLAG immunoprecipitation (IP). IP values from Control (wildtype) and *Fam209*$^{Flag/Flag}$ are presented as normalized total spectra.

Click here to download Table S2

Table S3. Mass spectrometry results of the third FAM209-FLAG immunoprecipitation (IP). IP values from Control (wildtype) and *Fam209*$^{Flag/Flag}$ are presented as normalized total spectra.

Click here to download Table S3

Table S4. Mass spectrometry results from DPY19L2 immunoprecipitation (IP). IP values from Control and *Fam209*$^{+1/+1}$ are presented as normalized total spectra

Click here to download Table S4

Table S5. Primers used in this study.

**RT PCR**				
mouse *Fam209*				
Forward	TGCCTGTTCTTGTCTCTGTG			
Reverse	TCACCAATTCCATCTCGAGC			
***Fam209* -3 +1 allele**				
gRNA	GTGCCTTGCGGCGGACACTTC	PAM	CGG	
Genotyping Primers				
Forward	AAGAATTCAAGGCATCACAATGACTG			
Reverse	AAGCTAGCGCAACTTCACCTAGCTATC			
Sequencing	AAGAATTCAAGGCATCACAATGACTG			
***Fam209*-FLAG Knockin**				
Fam209-FLAG gRNA	GAGGAGGACTCTGAGTGAAG	PAM:	AGG	
Fam209-FLAG sONA	ACCTGTACAATAATGTCACAATATATGAGATATGGGGGGAGGAGGACTCTGAGGACTACAAAGAC GATGACGACAAGTGAAGAGGTTTATAGTAAAGTATAAGAAAAGACAAGTATTGACAGACAGTATC			
Genotyping				
Forward	CATCCTCCTGGCCTCCGA			
Reverse	GAGGGGAGAAAGAGCTCACG			
Sequencing	CATCCTCCTGGCCTCCGA			
***Spaca9* KO**				
gRNA 1	CTTCTGCTCGATGCTGCGGA	PAM	GGG	
gRNA 2	GATTTTGTCGGGTAAATCCC	PAM	AGG	
Genotyping KO allele				
Forward	GGGTATACTCTGTGATCTGG			
Reverse	CTGCCCATCTTAACAGCAGG			
Genotyping WT allele				
Forward	GGGTATACTCTGTGATCTGG			
Reverse	CTGCCCATCTTAACAGCAGG			

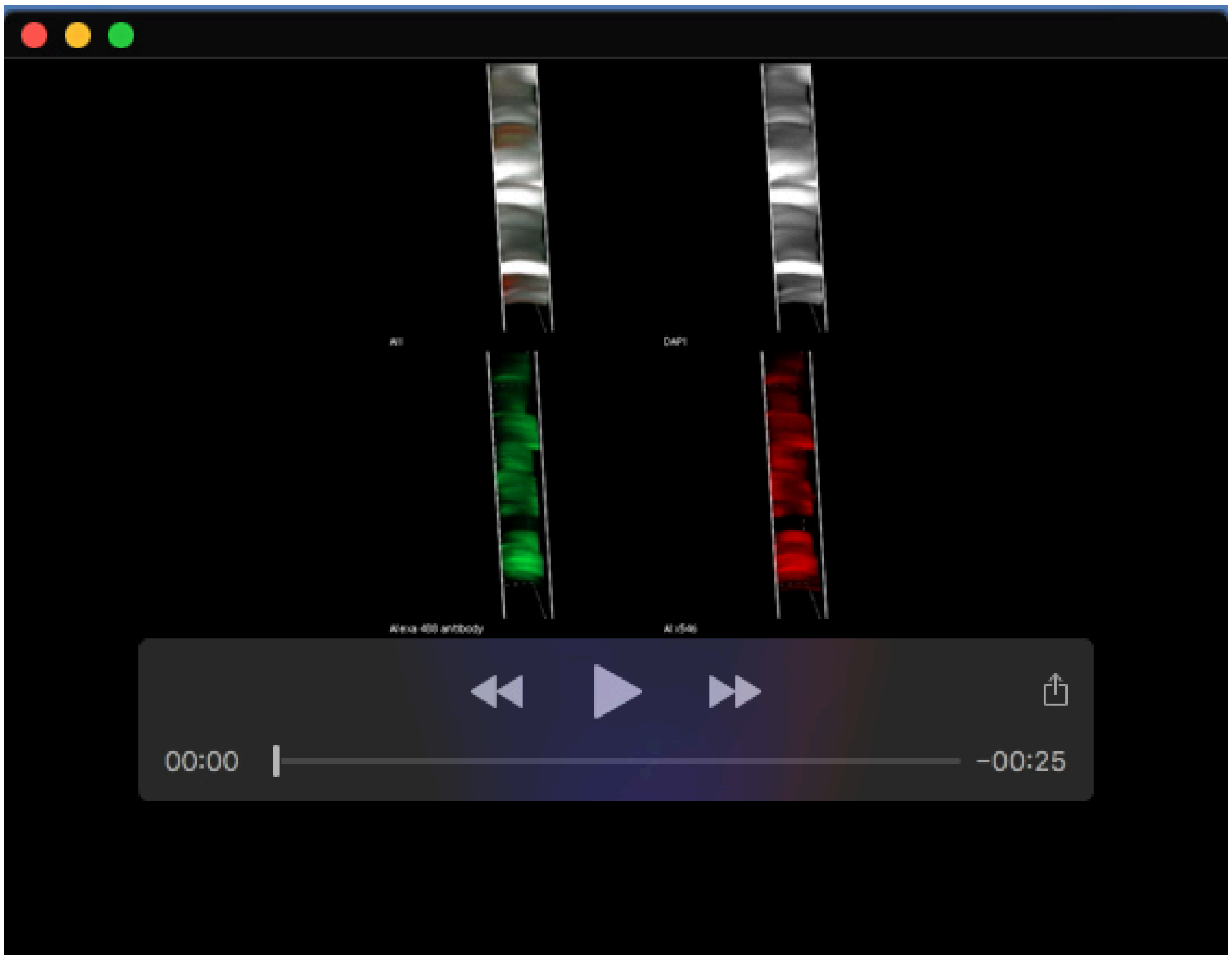

Movie 1. 3D reconstruction of Figure S4C. FAM209-FLAG (green), SPACA1 (red), Hoechst33342 (white) localization in spermatids.

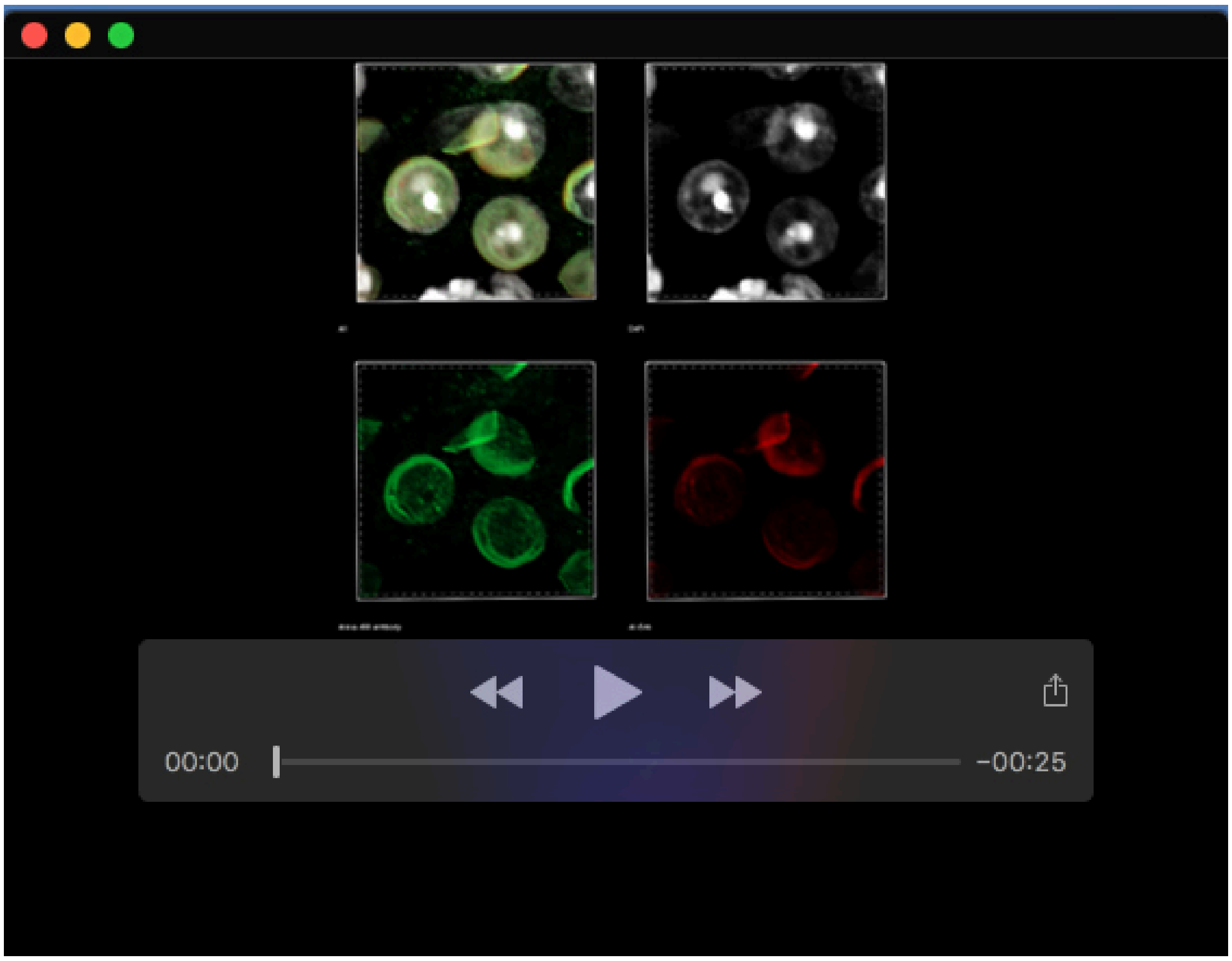

Movie 2. 3D reconstruction of Figure S4D. FAM209-FLAG (green), IZUMO1 (red), Hoechst33342 (white) localization in spermatids.

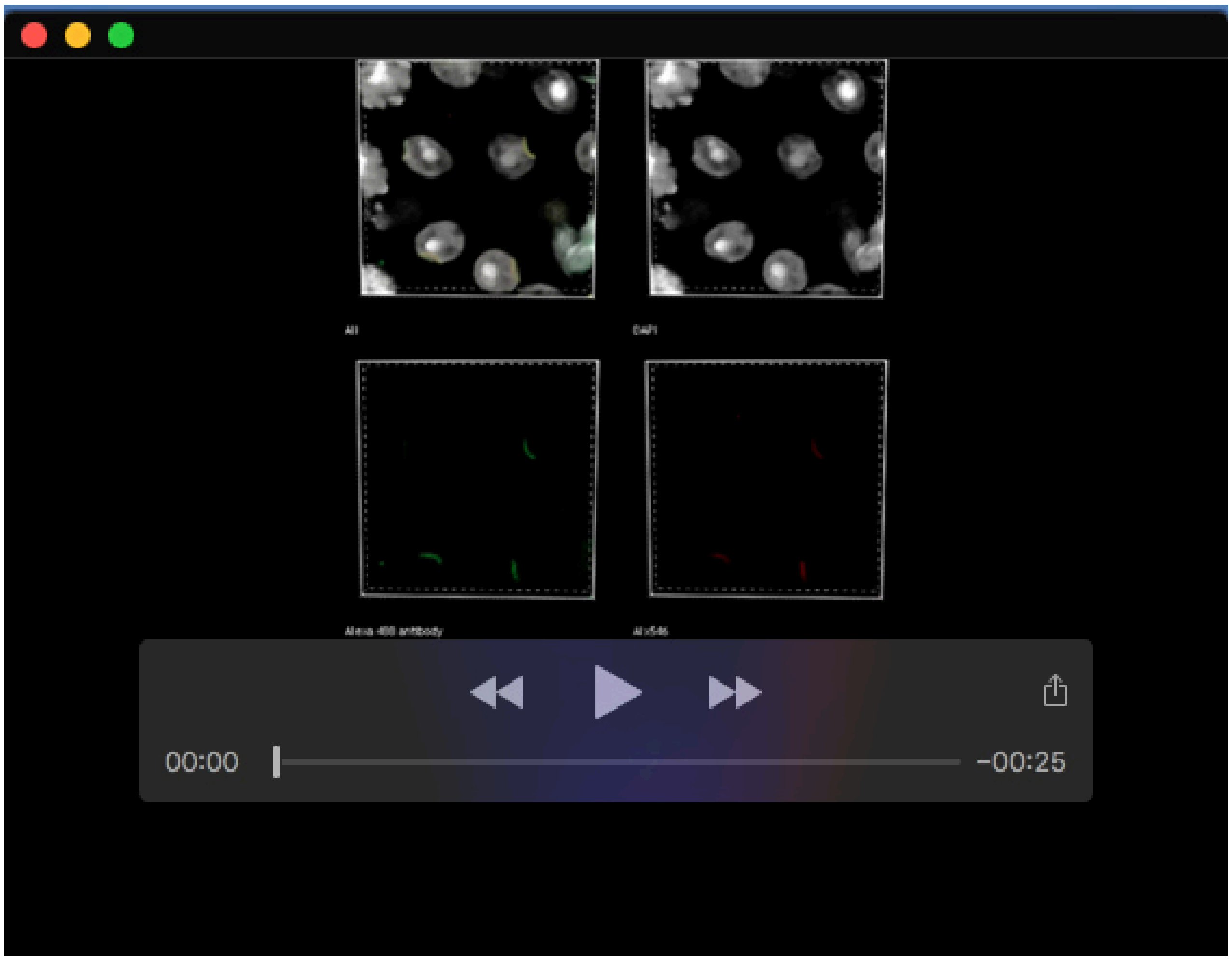

Movie 3. 3D reconstruction of Figure 6 Stage II-III. FAM209-FLAG (green), DPY19L2 (red), Hoechst33342 (white) localization in round spermatids.

Journal of Cell Science • Supplementary information

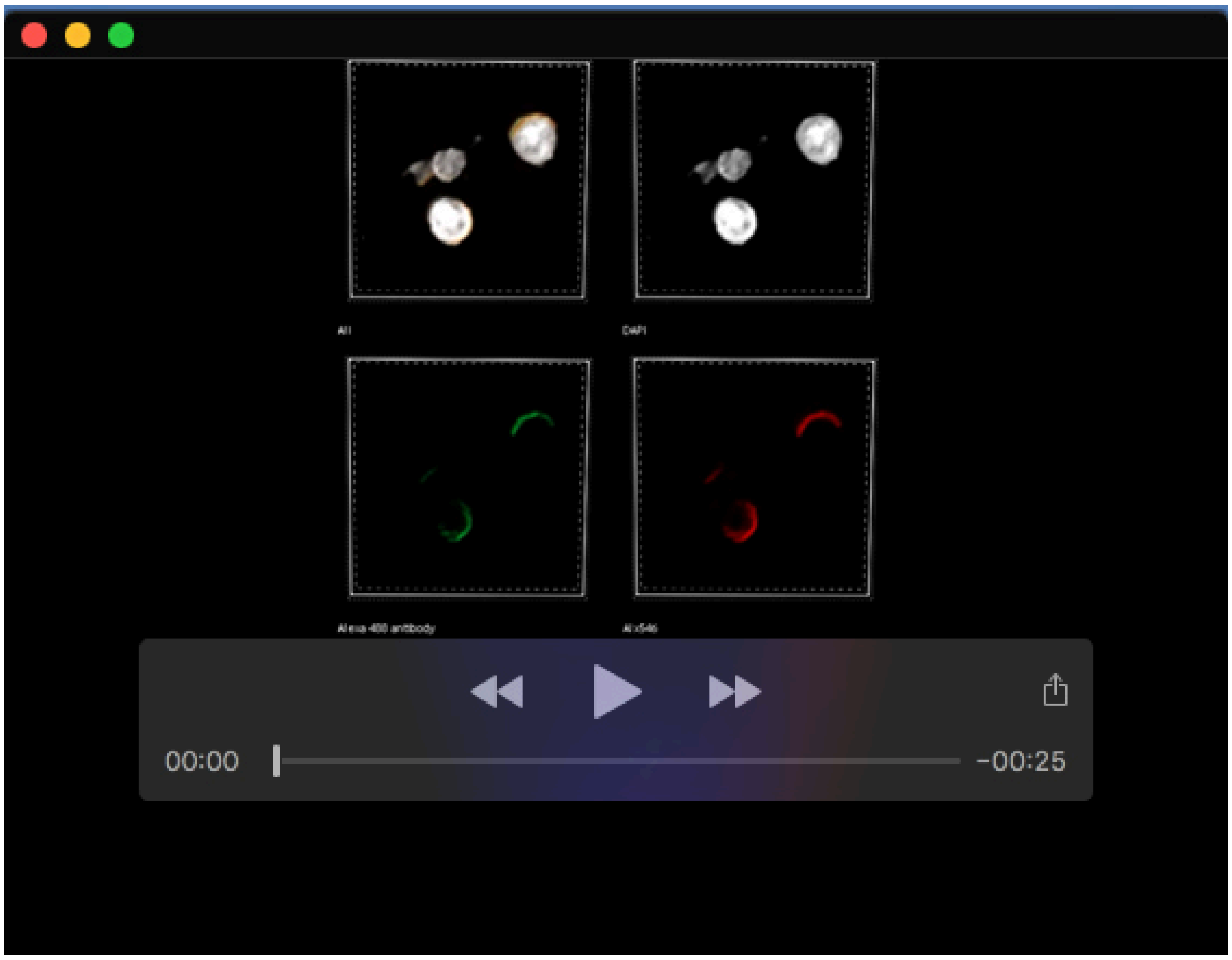

Movie 4. 3D reconstruction of Figure 6 Stage VI-VIII. FAM209-FLAG (green), DPY19L2 (red), Hoechst33342 (white) localization in round spermatids.
